# Study on Fire Temperature Field in Small-Section Steel-Shell Concrete Immersed Tube Tunnel Structure Based on CFD-FEM Method

**DOI:** 10.3390/ma18010187

**Published:** 2025-01-04

**Authors:** Bei Zhao, Baochao Xie, Zhisheng Xu, Feifan Wang, Yifan Gao

**Affiliations:** School of Civil Engineering, Central South University, Changsha 410075, China; beizhao1206@163.com (B.Z.); zhshxu@csu.edu.cn (Z.X.); feifanwang@csu.edu.cn (F.W.); gaoyifan@csu.edu.cn (Y.G.)

**Keywords:** small-section steel-shell concrete tunnel structure, CFD-FEM, heat transfer, temperature distribution, tunnel fire

## Abstract

Small-section steel-shell concrete immersed tube tunnels are intended for minibuses and have a low fire heat release rate. Standard fire rise curves do not apply to such tunnels. In this study, a coupled method of computational fluid dynamics (CFD) and the finite element method (FEM) was used to simulate the structural temperature distribution in tunnels. Firstly, a tunnel fire dynamics model was established to obtain the inhomogeneous temperature field during tunnel fires. Subsequently, a three-dimensional heat transfer analysis model for the tunnel tube section was established to simulate the temperature transfer characteristics of the tunnel structure with and without fire protection measures under different types of vehicle fires. This study showed that because steel has a higher thermal conductivity, at the same depth, the temperatures were the highest in T-ribs, followed by partitions, and the lowest in concrete; however, the steel components inside the tunnel minimally affected the tunnel temperature. Without fire protection, the steel shell’s surface temperature exceeded 300 °C in as little as 500 s. Temperature’s primary impact on the tunnel’s steel structure was within 30 cm of the steel shell’s surface, and on concrete, it was within 20 cm. The greatest temperature difference between the partition and concrete occurred 10 cm from the steel shell’s surface. These results fill the knowledge gap on heat transfer in these tunnels and have positive practical significance for the fire resistance design of tunnels.

## 1. Introduction

Immersed tube tunnels are mainly categorized into reinforced concrete immersed tube tunnels and steel-shell concrete immersed tube tunnels [1]. Currently, most of the immersed tube tunnels constructed in China are reinforced concrete structures, the most representative of which is the immersed tube tunnel of the Hong Kong–Zhuhai–Macao Bridge project. Although the cost of reinforced concrete structures is low and the design method and construction technology are relatively mature, under the requirements of high water pressure, large sections, high durability, and uneven settlement, it is still necessary to focus on solving the cracking problem of reinforced concrete structures, which is a significant limitation of the ultimate clear span of the immersed tube tunnel section. As a new type of tunnel structure form, steel-shell structure immersed tube tunnels, with their strong seismic resistance and good structural stability, are suitable for assembly-type fine processing and manufacturing, which is conducive to shortening the construction period and achieving better economic benefits and represents the world’s highest level and development direction of the construction of underwater immersed tube tunnels [2,3]. The development of six steel–concrete–steel structure immersed tube tunnels has been completed worldwide [4]. China constructed the Shenzhen–Zhongshan Access underwater part of the immersed tube section, which also adopted this structural form. China’s future construction on the Linjiang Avenue–Reading River Road tunnel will also adopt this structural form.

Although the construction of steel-shelled concrete immersed tube tunnels is progressing rapidly, few studies have been conducted on the technology related to this structural form. Current research on fires in immersed tube tunnels mainly focuses on fires under reinforced concrete structures, and the main research methods are field tests, numerical simulations, and theoretical analyses. In a temperature study on ordinary reinforced concrete tunnel structures, T. Ring [5,6] conducted a thermal coupling test of a foot-scale model with a local urban immersed tube tunnel as the research object, determined the warming and deformation of each part of the model, and observed, evaluated, and analyzed experimental phenomena that occurred during the fire. Du et al. [7] developed a 1:10 tunnel fire model and measured the surface and internal temperatures of the concrete lining during a fire test. Kim et al. [8] conducted a fire resistance test under the RABT fire warming curve for a reinforced concrete structure tunnel, investigated the peak temperature of the concrete surface of the tunnel structure, and proposed a desirable minimum coating thickness for tunnel concrete. Duan et al. [9,10] constructed a 1:5 immersed tube model tunnel and conducted fire tests of the immersed tube tunnel under the protection of fire-resistant coatings. During the fire test, the fire temperature, tunnel temperature, and tunnel deformation were recorded, and the thermal insulation performance of the fireproof coating was investigated. Guo et al. [11], with the Hong Kong–Zhuhai–Macao immersed tube tunnel as the basis, used a fire test, a theoretical analysis, a numerical simulation, etc., and conducted analyses with or without the thermal insulation of the temperature field distribution law and the change rule of structural thermal stress. Yan [12] investigated the temperature distribution of steel plates and concrete in tunnels under standard warming curves by using numerical simulation. Lin et al. [13] took concrete immersed tube tunnels as the object of study and carried out a 1:5 large-scale fire test to study the model’s temperature distribution, spalling degree, and crack distribution. Wang et al. [14] used fire dynamics simulation software and structural analysis software to analyze the temperature field and structural response law of immersed tube tunnels without fire protection measures and studied fire protection effects and fire protection schemes through a high-temperature test on fire protection materials.

In a temperature study of steel-shell concrete tunnel structures, Matsumoto et al. [15], relying on the Kobe Harbor Island Tunnel, designed two fire protection schemes of fireproof boards and fireproof coatings and investigated the surface of the steel shell and the internal temperature change law of the structure by using physical model tests and numerical simulations. Currently, Chinese research on the thermal response of steel-shell concrete immersed tube tunnel structures mainly focuses on studying the Shenzhen–Zhongshan Access. Zhang and Cao et al. [16,17] simulated the change law of the temperature field of steel-shell concrete composite structures under the RABT curve by using ANSYS and analyzed the temperature change in the structure under different heights and fire times in detail. Cao et al. [18] carried out a scaled-down fire resistance test on a steel-shell concrete immersed tube structure and compared the results of numerical simulations and physical model tests to reveal the temperature transfer mechanism inside this combined structure at high fire temperatures and analyzed the temperature transfer law of the combined structure at different depth measurement points. Cao et al. [19] used the finite element method to establish three-dimensional finite element models of steel–concrete composite structures under different fire conditions in tunnels and investigated the fire resistance of steel–concrete–steel (SCS) structures and reinforced concrete (RC) structures of mega-span immersed tube tunnels. Li et al. [20] carried out local full-size component tests and established finite element models of steel-shell concrete structural components to study the internal temperature distribution law of the pipe section structure and the temperature change state of the bottom steel shell under different fire-resistant configurations in the high temperature of fire. However, there is a big difference between the small cross-section of a steel-shell concrete immersed tube tunnel and the Shenzhen–Zhongshan Access regarding passing cars, cross-section size, and design fire heat release rate, so the reference value is limited.

Due to the unique characteristics of the tunnel structure, it is difficult to quickly control the fire once it occurs during tunnel operation, which will cause irreversible damage to the tunnel structure. Research on the temperature field of small-section steel-shell concrete immersed tube tunnel structures is still lacking. Moreover, most previous numerical studies on tunnel fire responses were based on various standard temperature curves, which failed to consider the actual situation of tunnel fires. Therefore, studying the temperature transfer law of a structure under fire is necessary for the new small-section steel-shell concrete composite structure of immersed tube tunnels. Based on engineering practice and considering vehicle fires of different types, this paper uses the CFD-FEM coupled thermal analysis method to derive the temperature transfer law and temperature distribution under fire conditions in the tunnel structure. This fills the knowledge gap in studying the structural temperature field of this type of tunnel under different fire scenarios and provides an essential reference for studying the fire resistance of this type of tunnel.

## 2. Materials and Methods

### 2.1. CFD-FEM Coupled Method for Thermal Analysis

**Figure 1:**
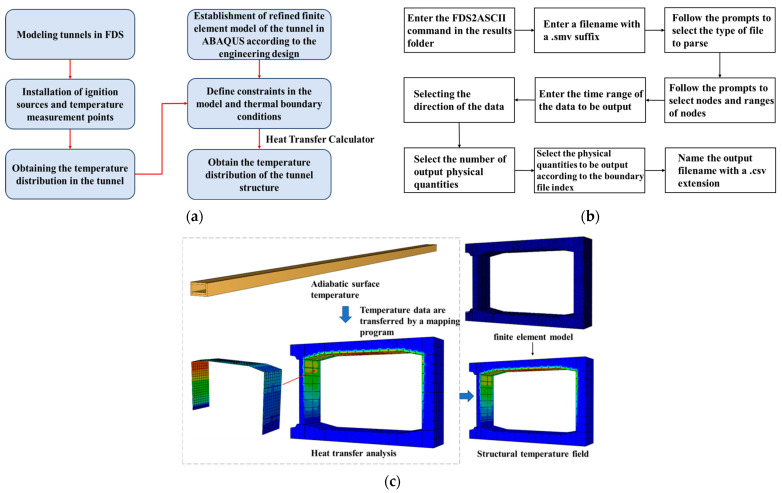
Schematic diagram of CFD-FEM. (**a**) Analysis process, (**b**) steps in the mapping procedure to retrieve the adiabatic surface temperature, and (**c**) step visualization.

The analysis process of the coupled computational fluid dynamics and finite element method (CFD-FEM) method is shown in Figure 1a. Firstly, the fire temperature field model was established. Secondly, the temperature distribution data obtained from the FDS simulation were imported into the tunnel’s established 3D refined thermal analysis model. Finally, the thermal analysis solver was executed to obtain the temperature distribution within the tunnel structure through calculation, as shown in Figure 1c, and the mapping procedure to retrieve the adiabatic surface temperature is shown in Figure 1b.

### 2.2. Fire Temperature Field Model

**Figure 2:**
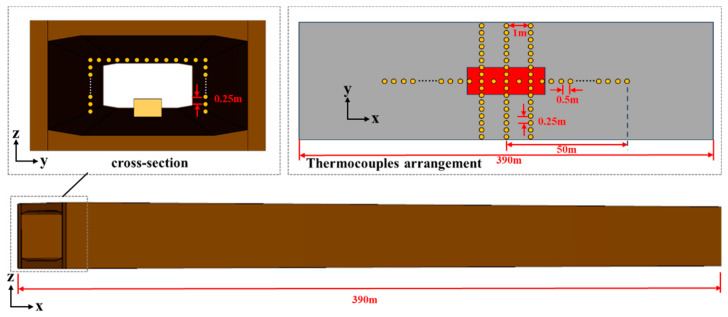
Model setting and thermocouple arrangement.

The two caverns of the small-section tunnel are separated by an intermediate corridor with fire separation so that the two caverns do not affect each other in the event of a fire, and only the situation within a single cavern is considered in the analysis. The width of the carriageway level is 8.95 m, and the clear height is 4.3 m. In the simulation, the fireproof boards were single-layer calcium silicate boards, the ignition source was heptane, and the thermal parameters of the tunnel structure are shown in Table 1. A numerical model of the tunnel was created by using the fire dynamics software Fire Dynamics Simulator (FDS) (version 6.7.5), which is developed by the National Institute of Standards and Technology in the USA. The tunnel model was 390 m long, with open boundaries at both ends. The ambient temperature and ambient pressure were 293 K and 101.32 kPa, respectively. The tunnel model and thermocouple layout are shown in Figure 2.

The tunnel is a small-section road tunnel that allows only minibuses and buses to pass through. According to the standard [21], following the most unfavorable principle, the fire heat release rates of minibuses, vans, and buses were selected as 8 MW, 15 MW, and 30 MW, respectively. According to previous research [22,23,24,25] and considering the effect of the fireproof board, which was 25 mm thick, the set-up working conditions of this study are shown explicitly in Table 2. Therefore, six fire source powers were selected in this study, which were 8 MW, 15 MW, 30 MW, 16 MW, 24 MW, and 40 MW, and the t^2^ growth model was used. The fire development was assumed to be super-rapid, and the fire growth factor was 0.1876 kW/s^2^.

**Figure 3:**
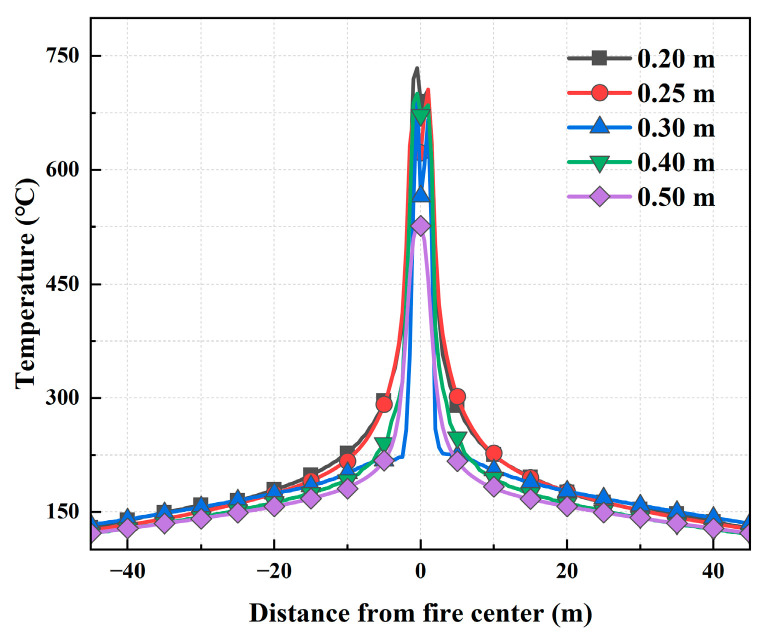
Temperature distribution in the tunnel with different grid sizes.

In FDS simulations, the computational accuracy is closely related to the grid quality. A related study [26] showed that the reasonableness of the simulation results could be ensured when the ratio of the fire source characteristic diameter to the mesh size (*δ*) was between 4 and 16. Equation (1) shows the characteristic diameter of the fire source [27]:

(1) $$D^{*}={\left(\frac{\dot{Q}}{\rho_{\infty }c_{p}T_{\infty }\sqrt{g}}\right)}^{\frac{2}{5}}$$

where D* is the characteristic diameter of the fire, *ρ* and *T* are the density of the air and the ambient air temperature, respectively, *g* is the acceleration of gravity, $\dot{Q}$ is the total heat release rate, kW, and *c_p_* is the specific heat of ambient air. From the working conditions in this paper, the grid size *δ* is set in the range of 0.14 to 1.05 m. To balance the relationship between the accuracy of the calculation results and the calculation time, the grid sensitivity analysis was carried out by selecting grid sizes of 0.20 m, 0.25 m, 0.30 m, 0.40 m, and 0.50 m for the HRR of 8 MW as an example, and the longitudinal temperatures at the central axis were used as the grid sensitivity analysis index, as shown in Figure 3. When the grid size *δ* was 0.30 m, the calculation time could be saved while meeting the accuracy of the numerical simulation.

### 2.3. Heat Transfer Analysis Model

**Figure 4:**
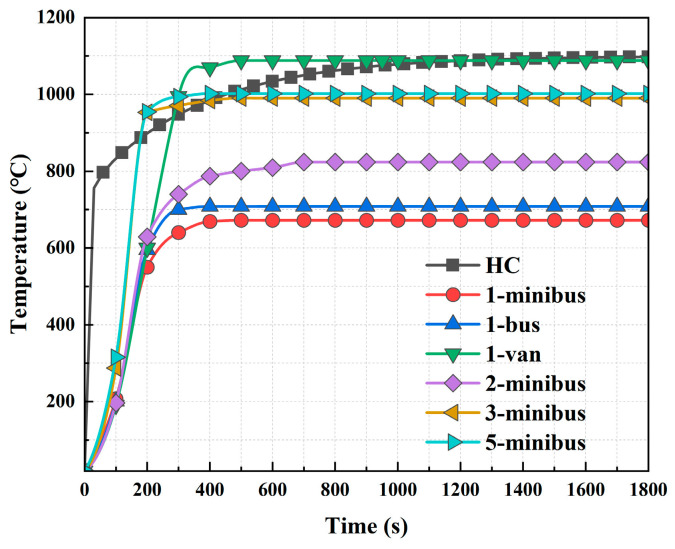
Fire heating curve beneath the ceiling.

**Figure 5:**
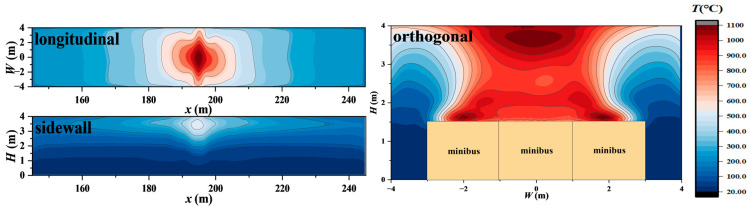
Contour of smoke temperature distribution during combustion of 3 minibuses.

According to the recommendation of the GB50016-2014 (2018 edition) Code for the fire protection design of buildings [28], tunnels with a closed section length between 500 m and 1500 m are categorized as Class III tunnels, and the fire heating curve should adopt the HC standard heating curve. In this classification, small-section steel-shell concrete immersed tube tunnels are classified as Class III tunnels, and the HC curve should be used. It is still to be confirmed via further analysis whether the provisions of the Chinese domestic standard [28] on the adoption of the HC temperature curve for Class III tunnels apply to small cross-section, minibus-only tunnels. On the one hand, the power of the fire source during a minibus fire is small, and the heat released per unit of time is less, which is favorable to structural fire resistance; on the other hand, the tunnel section is small, and it is easy for the heat to accumulate, which is unfavorable to structural fire resistance. Therefore, in addition to using the HC curve, the spatial smoke temperatures at each location were obtained based on the study of the temperature field of different types of vehicle fires in the tunnel in Section 2.2. The simulated adiabatic surface temperatures were then applied to the finite element model of the tunnel sections. Temperature loading was applied to the tunnel section through CFD-FEM coupling, which is more realistic than standard temperature rise curves. Therefore, in the structural fire resistance analysis, a comparative study was mainly carried out between the HC standard temperature rise curve and the structural temperature field simulated under the temperature field of an inhomogeneous fire, using the finite element model ABAQUS (version 2023), setting up the heat transfer conditions, as shown in Table 3. The temperature rise curves at the highest temperatures of different types of vehicle fires below the ceiling are shown in Figure 4. Instead of this rise curve, the fire temperature field obtained from the simulation was used in the heat transfer analysis, as shown in Figure 5, and the fire temperature distribution obtained from the simulation was inhomogeneous.

**Figure 6:**
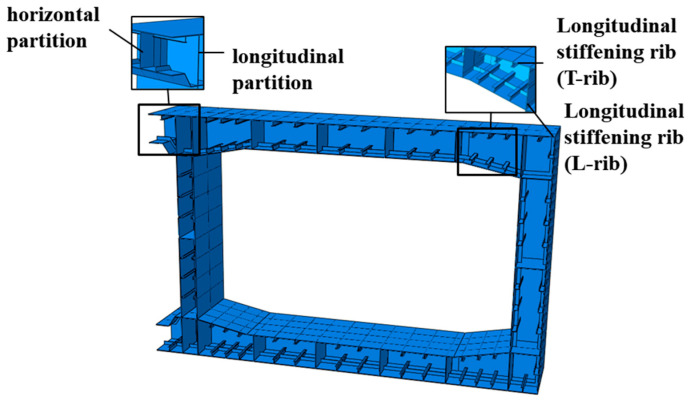
Numerical modeling of the steel shell.

Relying on the project, a three-dimensional numerical heat transfer model of the tunnel was established, as shown in Figure 6. The horizontal partition was spaced 2 m along the longitudinal direction of the tunnel, and the model was established by taking 1.0 m on each side with the horizontal partition as the center. At the same time, since the structure also had symmetry in the transverse direction and the fire high-temperature transmission distance was limited, the model was established as a semi-structure in the transverse direction. The “Tie” was set up between the steel plate and the concrete. In this way, when the steel–concrete composite structure was subjected to fire, the heat could be transferred from the steel plate to the concrete, transverse, and longitudinal partitions and ribs, effectively simulating the heat transfer inside the member when a fire occurred. DC3D8 cells were used for meshing for heat transfer calculation.

This study selected concrete of strength class C50 and Q355 steel based on the materials used in small cross-section tunnels. Steel’s thermal conductivity and specific heat capacity were determined according to Eurocode [29], and its density was taken as a constant value of 7850 kg/m^3^. The thermal conductivity and specific heat capacity of concrete were also adopted from Eurocode [29], and its density was taken as a fixed value of 2400 kg/m^3^. The fireproof board was a single-layer calcium silicate fireproof board. According to the characteristics of the product and the construction process, its thermal conductivity was taken as 0.242 W/(m·K), the specific heat capacity was taken as 800 J/(kg·K), and the density was 1086 kg/m^3^. The selection of temperature loads was based on the HC curve and the study of the temperature field of different types of vehicle fires in tunnels in Section 2.2, and the tunnel tube section structure was treated as a Type III boundary condition. The convective heat transfer coefficient was 50 W/(m^2^·K) [30], the Stephen Boltzmann constant was 5.67 × 10^−8^ W/(m^2^·K^4^), and the ambient temperature was 20 °C.

**Figure 7:**
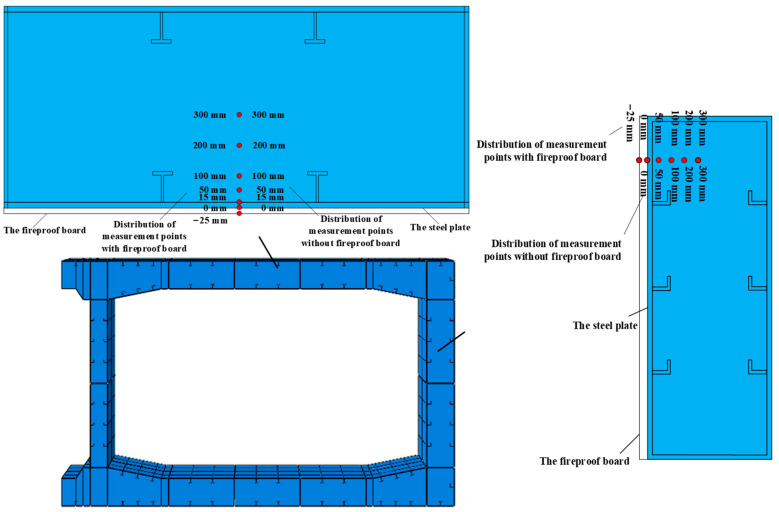
Distribution of measurement points inside the member.

The tunnel ceiling measurement points were arranged as follows: When there was no fireproof board, measurement points were set vertically from the center of the steel plate bottom at 0 mm, 15 mm, 50 mm, 100 mm, 200 mm, and 300 mm from the outer surface of the steel shell. When there was a fireproof board, the measuring points were set vertically from the middle of the bottom surface of the fireproof board. They were −25 mm, 0 mm, 15 mm, 50 mm, 100 mm, 200 mm, and 300 mm from the outer surface of the steel shell. The arrangement of measurement points on the side walls was similar to that of the tunnel ceiling, and the specific arrangement is shown in Figure 7.

### 2.4. Numerical Model Validation

**Figure 8:**
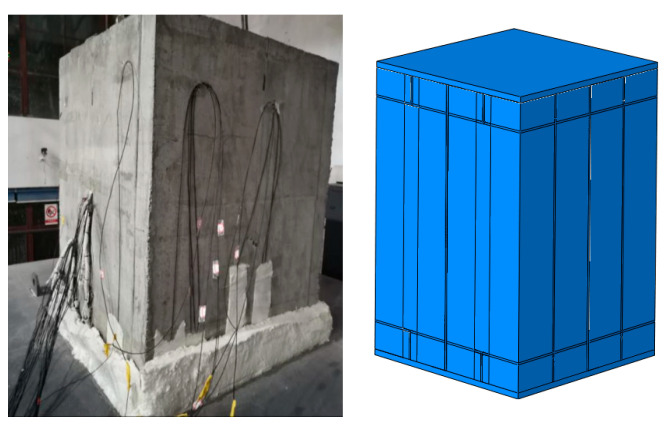
The test component and the simulation model.

**Figure 9:**
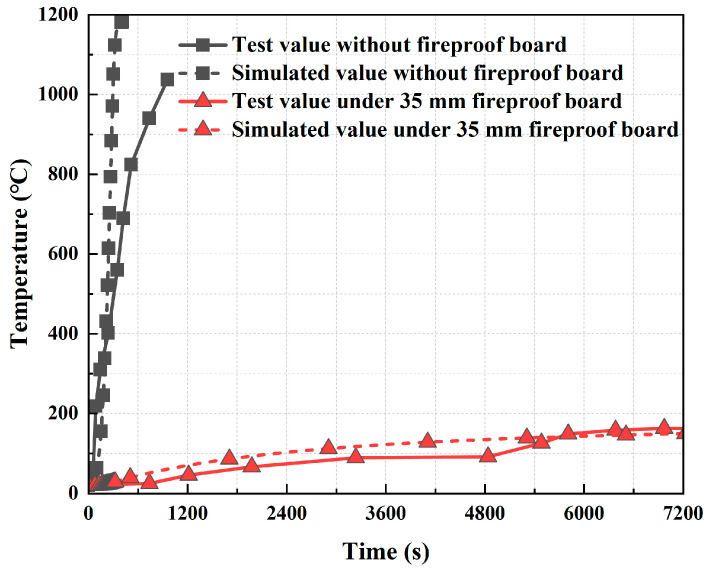
Comparison of measured and simulated temperatures at the center of the base of the steel shell under the RABT curve.

There are currently no tests related to small-section steel-shell concrete immersed tube tunnels for us to verify. During a fire, the heat transfer of this structure is from the steel to the concrete, following the same processes and mechanisms as those of other steel-shell concrete structures. Therefore, we looked for experiments [31] on steel–concrete composite structures to verify our model. We used the method of establishing a structural model of a small-section steel-shell concrete immersed tube tunnel. The test component and the simulation model are shown in Figure 8. The comparison of the measured and simulated temperatures at the center of the base of the structural steel casing under the RABT curve is shown in Figure 9. As can be seen from the figure, the simulation results agree well with the test results, indicating that the established model is accurate.

## 3. Results and Discussion

### 3.1. Characteristics of Temperature Distribution in the Tunnel Tube Section Structure

**Figure 10:**
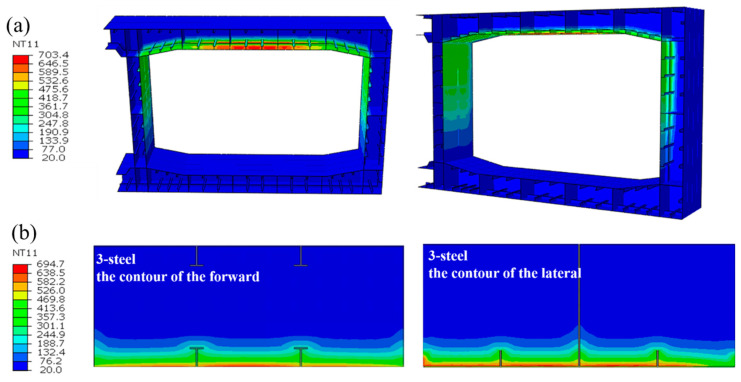
The contour of the structural temperature field distribution at 7200 s. (**a**) Contour of steel-shell structure temperature distribution at 7200 s. (**b**) Contour of concrete temperature distribution at 7200 s.

Figure 10 shows the contour of the structural temperature field distribution at 7200 s under this condition, considering the combustion of three minibuses as an example. There was a significant temperature gradient within the tunnel tube section structure, with the temperature increasing closer to the outer surface of the tunnel steel shell. Without fire protection, the temperature of the steel-shell structure can reach 703.4 °C, the temperature of the concrete structure can reach 694.7 °C, and the temperature of the concrete structure away from the outer surface of the steel shell is only 20 °C. The temperature is distributed in a “convex” shape within the compartment enclosed by horizontal and longitudinal partitions. The temperature has a greater depth of influence at the T-rib and horizontal and longitudinal partitions and the lowest depth of influence at the concrete. This is mainly because the longitudinal and horizontal partitions, T-ribs, and horizontal ribs are made of steel, which has a higher thermal conductivity than concrete. Therefore, the temperature transfer along the steel plate is significantly faster than that along the concrete during the temperature transfer process.

**Figure 11:**
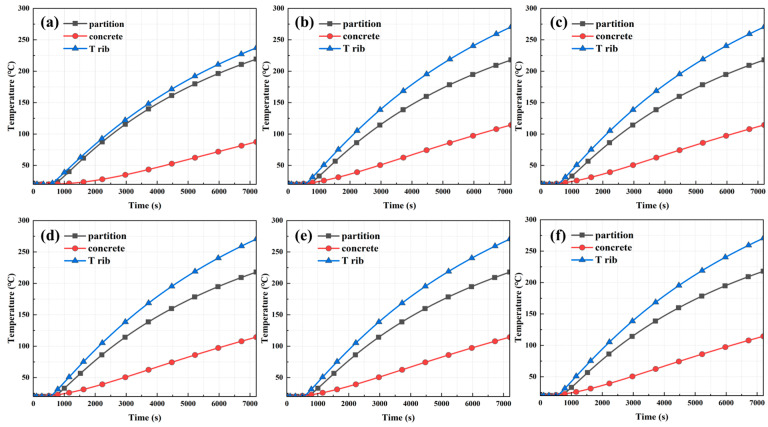
Temperature change rule of measuring points at different positions with time: (**a**) 1-minibus, (**b**) 1-bus, (**c**) 1-van, (**d**) 2-minibus, (**e**) 3-minibus, and (**f**) 5-minibus.

To investigate the variability in temperature transfer at different locations at the same depth inside the tunnel tube structure, measurement points were set up on the tunnel ceiling at different locations (partitions, concrete, and T-ribs) 100 mm from the outer surface of the steel shell. The results of the temperature change rule of measuring points with time under different types of vehicle fires are shown in Figure 11. The temperature increase trends at the T-ribs and the partitions were highly similar. Simultaneously, the temperature at the T-ribs was slightly higher than that at the partition board, which may be related to the lower height of the T-ribs. Maximum temperatures of up to 328 °C at the T-rib and 285 °C at the partition were observed for different types of vehicle fires. At the same depth, the rate of temperature increase at the concrete was significantly lower than that at the T-ribs and partitions, with a maximum temperature of 146 °C. This result indicated a significant difference in the temperature response of the different parts of the tunnel tube section structure under fire conditions. The structural temperatures were highest at the T-rib, second highest at the horizontal and longitudinal partitions, and lowest at the concrete at the same depth.

**Figure 12:**
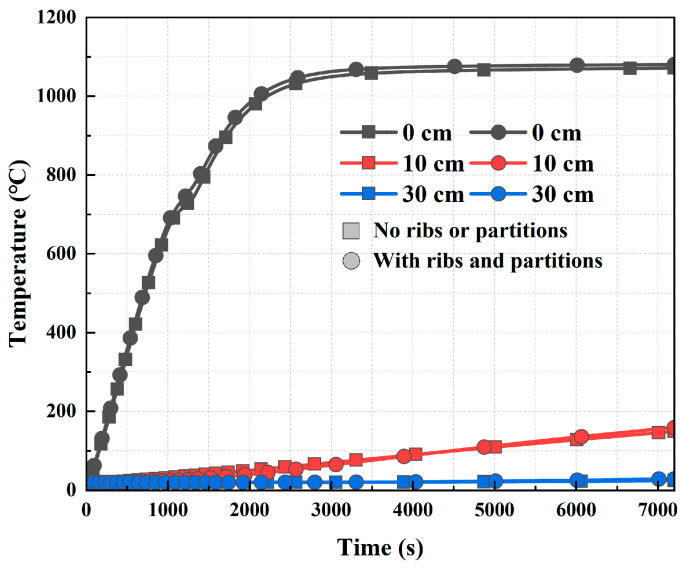
Comparison of temperature distribution at the same depth.

To investigate the effect of internal stiffening of ribs and partitions on the temperature distribution of tunnel structures in the tunnel tube section structure, HC standard curve conditions were selected to compare and analyze the temperature data at the same locations within the tunnel structure with and without stiffening ribs and partitions, as shown in Figure 12. The temperature gap was small when stiffening ribs and partitions were present within the structure and when there were no stiffening ribs and partitions. At different distances from the outer surface of the steel shell, the temperature difference was only 10 °C. This result showed that although steel structures heated rapidly in a fire, their effect on the overall structural temperature of the tunnel was relatively limited, and the influence of steel components inside the tunnel on the overall structural temperature of the tunnel was small.

### 3.2. Temperature Distribution Law of Tube Section Structure with Depth

**Figure 13:**
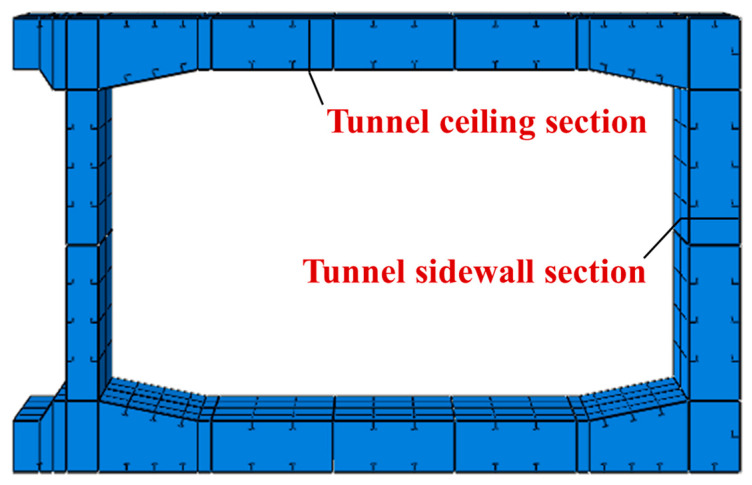
Schematic diagram of the selected section of the structure.

In the immersed tube tunnel cross-section, to illustrate the temperature distribution in each part of the cross-section, the ceiling section and sidewall section shown in Figure 13 were selected to analyze the simulation results.

#### 3.2.1. Analysis of the Temperature Field Under Conditions Without Fire Protection Measures

**Figure 14:**
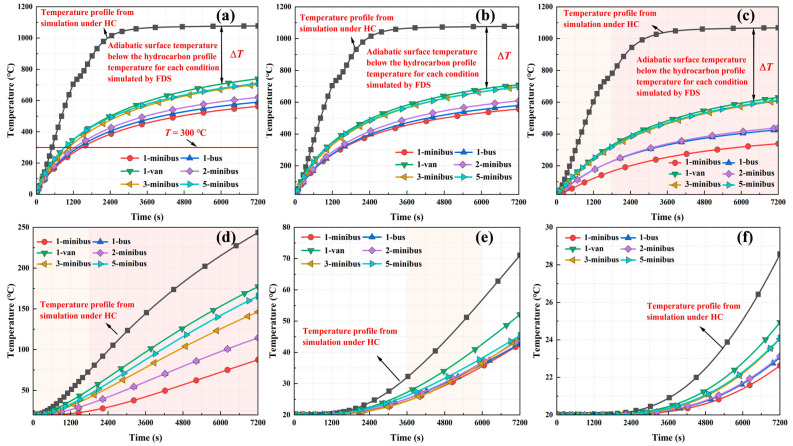
Temperature change law with time at different depths of the tunnel ceiling when no fire protection measures were taken: (**a**) 0 cm, (**b**) 1.5 cm (concrete surface), (**c**) 5 cm, (**d**) 10 cm, (**e**) 20 cm, and (**f**) 30 cm.

**Figure 15:**
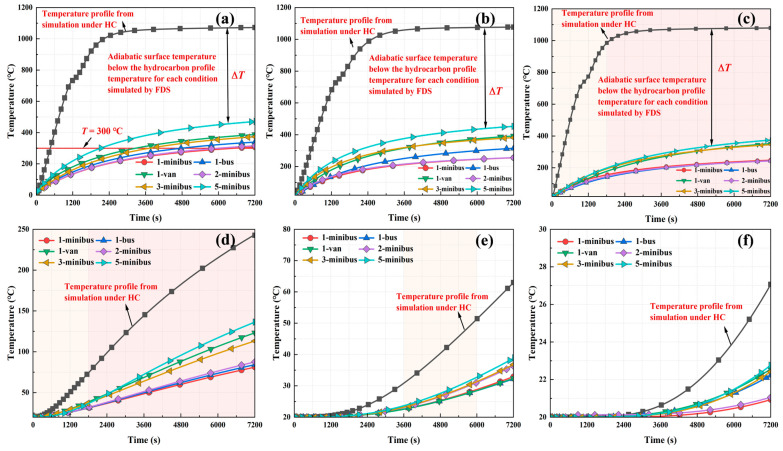
Temperature change law with time at different depths of the tunnel sidewall when no fire protection measures were taken: (**a**) 0 cm, (**b**) 1.5 cm (concrete surface), (**c**) 5 cm, (**d**) 10 cm, (**e**) 20 cm, and (**f**) 30 cm.

The temperature distributions of the tunnel ceiling and sidewalls at different distances from the steel shell’s outer surface without fire protection conditions are shown in Figure 14 and Figure 15, respectively. Under the HC curve and various vehicle fire scenarios, the increasing temperature trend at depths of up to 5 cm from the tunnel steel-shell structure was consistent. Beyond a depth of 5 cm, the temperature increase was attenuated by thermal transfer delays, resulting in a diminished heating rate for all conditions. At a depth of 30 cm, the structural temperature converged with the ambient temperature. In summary, the temperature increase within the tunnel structure was predominantly confined to a 30 cm zone from the steel shell’s outer surface, with temperatures beyond this area nearing ambient levels.

In all scenarios, at depths of up to 5 cm from the tunnel steel-shell structure, temperatures increased rapidly, exhibiting marked temperature differences among conditions. For depths between 5 cm and 10 cm, structural temperatures rose notably within 30 min after the fire started. For depths between 10 cm and 20 cm, significant temperature increases occurred solely between 60 and 100 min after the fire started. Structural temperature changes were minimal at depths exceeding 20 cm. Moreover, in the case of a tunnel without fire protection, whether it was a single-vehicle spontaneous combustion or a multi-vehicle collision combustion condition, the temperature of the steel-shell surface at the ceiling and sidewall exceeded 300 °C, which is higher than the NFPA 502 fire protection temperature for steel-tunnel-lining surfaces. Assuming that 300 °C or higher is the high-temperature region, the depth of the high-temperature influence of the structure under the fires of different types of vehicles was approximately 5 to 10 cm from the outer surface of the steel shell.

#### 3.2.2. Analysis of the Temperature Field Under Fire Protection Conditions

**Figure 16:**
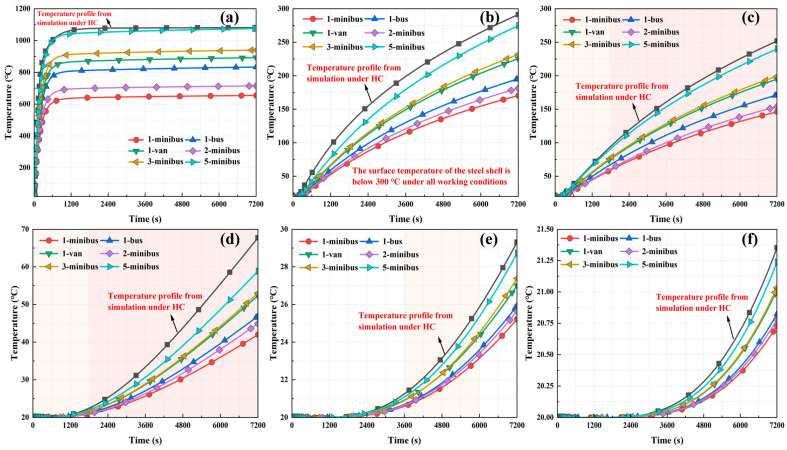
Temperature change law with time at different depths of the tunnel ceiling when fire protection measures were taken: (**a**) −2.5 cm, (**b**) 0 cm, (**c**) 5 cm, (**d**) 10 cm, (**e**) 20 cm, and (**f**) 30 cm.

**Figure 17:**
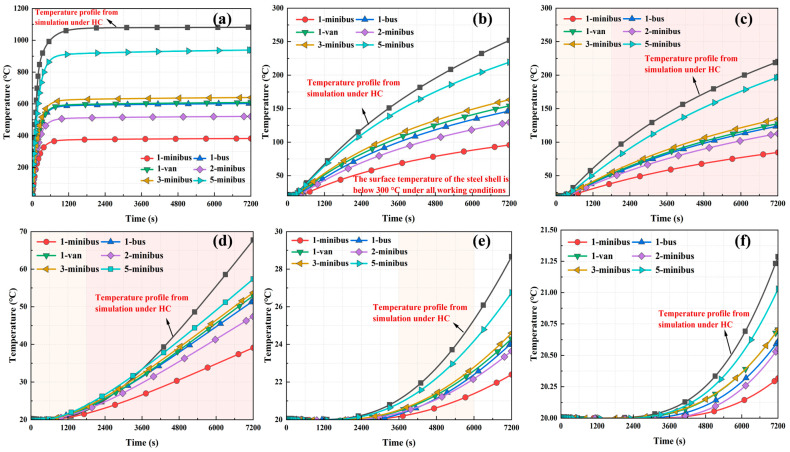
Temperature change law with time at different depths of the tunnel sidewall when fire protection measures were taken: (**a**) −2.5 cm, (**b**) 0 cm, (**c**) 5 cm, (**d**) 10 cm, (**e**) 20 cm, and (**f**) 30 cm.

The temperature distributions of the tunnel ceiling and sidewalls at different distances from the steel shell’s outer surface under fire protection conditions are shown in Figure 16 and Figure 17, respectively. Under the HC curve and various vehicle fire scenarios, the temperature increase trend at depths of up to 5 cm from the tunnel steel-shell structure was consistent. Beyond a depth of 5 cm, the temperature rise was attenuated by thermal transfer delays, resulting in a diminished heating rate for all conditions. At a depth of 30 cm, the structural temperature converged with the ambient temperature. In summary, the temperature rise within the tunnel structure was predominantly confined to a 30 cm zone from the steel shell’s outer surface, with temperatures beyond this area nearing ambient levels.

In all scenarios, at depths of up to 5 cm from the tunnel steel-shell structure, temperatures increased rapidly, exhibiting marked temperature differences among conditions. For depths between 5 cm and 10 cm, structural temperatures rose notably within 30 min after the fire started. For depths between 10 cm and 20 cm, significant temperature increases occurred solely between 60 and 100 min after the fire started. Structural temperature changes were minimal at depths exceeding 20 cm. The steel shell and concrete of the immersed tube tunnel heated up relatively slowly, attributed to the influence of fireproof boards. Taking the HC curve as an example, the steel-shell surface temperature reached approximately 300 °C solely after 4 h of combustion. Without fire protection, the temperature of the surface of the steel shell increased to 300 °C in approximately 500 s. This effect was due to fireproof boards effectively sequestering heat flux from direct contact with structural walls, thereby lowering the temperature at which the heat was conducted to the walls and substantially mitigating internal temperatures. In addition, the fireproof boards could effectively control the temperature of the steel shell under different types of vehicle fires to below 300 °C, which is lower than the NFPA 502 fire protection temperature for steel-tunnel-lining surfaces, while the structural temperature at the side walls was lower than that at the tunnel ceiling. It can be concluded that all seven working conditions with fire protection measures were in safe condition, and the single-layer calcium silicate board could effectively reduce the temperature of concrete and steel shells.

#### 3.2.3. Differences in Temperature Transfer Between Steel and Concrete

**Figure 18:**
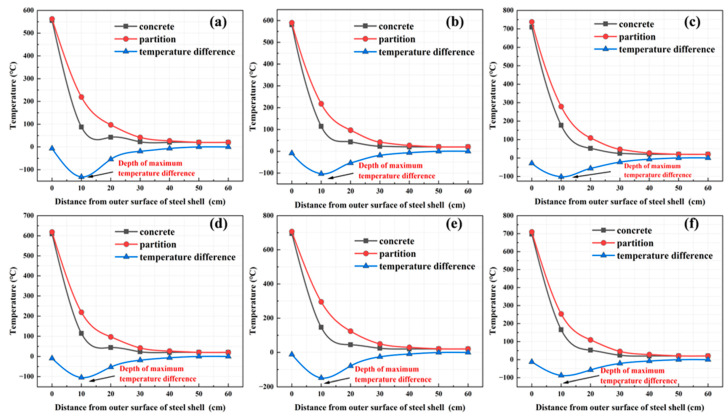
Comparison of temperature distribution between the partition and concrete at the same depth: (**a**) 1-minibus, (**b**) 1-bus, (**c**) 1-van, (**d**) 2-minibus, (**e**) 3-minibus, and (**f**) 5-minibus.

To further investigate the difference in temperature transfer between steel and concrete in small-section steel-shell concrete immersed tube tunnel structures, the maximum temperatures at the concrete and diaphragm at the same depth when the tunnel was not protected against fire were selected for comparative analysis, as shown in Figure 18. Temperature profiles under different types of vehicle fires showed the same trend: at the same depth, the temperature at the partition was higher than that of the concrete. This finding is consistent with the structural temperature distribution observed in the characteristics of the temperature field distribution in Section 3.1. This is mainly because partitions are made of steel, which has a higher thermal conductivity than concrete. The temperature decreased as the distance between the measuring point and the outer surface of the steel shell increased, and eventually, both leveled off. For the partition, the temperature was strongly influenced by the distance up to 30 cm from the outer surface of the steel shell, and the influence decreased after 30 cm. For concrete, the temperature effect was more significant at distances up to 20 cm. The temperature at the partitions was always higher than that at the concrete, and the temperature difference between the partitions and the concrete within 10–20 cm from the outer surface of the steel shell was maintained at about 100 °C. The maximum difference between the temperature at the partitions and that at the concrete was located 10 cm from the outer surface of the steel shell, and the maximum temperature difference was up to 149 °C.

## 4. Discussion

Most current research on the thermal response of steel-shell concrete structures is based on standard temperature rise curves [16,17,18,19,20]; however, due to the characteristics of small-section steel-shell concrete immersed tube tunnels, which include small sections, being dedicated to the passage of small passenger cars, and having low designed fire heat release rates, standard temperature rise curves do not apply to such tunnels. Therefore, we studied the temperature transfer law of the structure of small-section steel-shell concrete immersed tunnels.

Our study extended the analyses by Li and Cao et al. [16,17,18,19,20] regarding steel-shell concrete composite structures. We adopted a coupled CFD-FEM approach to further explore the temperature transfer laws in small-section steel-shell concrete immersed tube tunnels under various fire scenarios and revealed the significant differences in temperature transfer between steel and concrete, which can more accurately simulate real fire scenarios and significantly improve the structural temperature distribution prediction accuracy during a fire compared to the standard temperature profiles used in traditional fire response studies. The CFD-FEM methodology has the potential to be adapted to various tunnel configurations and sizes. The research found that the temperatures of the T-ribs and partitions within the tunnel exceeded those of the concrete, aligning with findings from Zhang (2023) [16], Cao (2020) [18], and Li (2022) [20]. Under different types of vehicle fires, the main effect of temperature on the steel structure of the tunnel was to a depth of 30 cm from the outer surface of the steel shell and on the concrete to a depth of 20 cm, which is different from their research results [18]. This was attributed to variations in the thermal properties of tunnel materials, fire scenario configurations, and structural dimensions. The maximum temperature of the structure under different types of vehicle fires was lower than the temperature under the HC curve, and the temperature of the steel shell generally exceeded 300 °C, exceeding the NFPA 502 [21] specified fire protection temperature for steel-tunnel-lining surfaces. This result has not been reported before. We studied the protective effect of a single-layer calcium silicate board on the tunnel structure. The research findings showed that the fireproof layer effectively blocked the contact between the high-temperature hot air flow from the fire and the structure of the tube section, reducing the impact of the high fire temperature on the internal temperature of the structure of the immersed tube tunnel section. The effect of heat insulation and temperature reduction was significant, which is consistent with the research results of Li (2022) [20].

These findings fill the knowledge gap on the structural temperature field of small-section steel-shell concrete immersed tube tunnels under different fire scenarios and provide an important reference for the study of the fire resistance of this type of tunnel. In this study, we chose to use standard values for the simulation analysis because we could not directly measure the specific parameters of the actual material. These standard values may deviate from the actual material parameters. We put much effort into building the model, considering various factors. Although our model is well designed, we know it lacks experimental verification. In the future, we will study more conditions by using a combination of experiments and numerical simulations.

## 5. Conclusions

In this study, a coupled CFD-FEM thermal analysis simulation method was used. Based on this method, the temperature transfer characteristics of the tunnel structure with and without fire protection measures under the HC curve and different types of vehicle fires were simulated and analyzed, and the following conclusions were drawn:

(1) Since the thermal conductivity of steel is higher than that of concrete at the same depth, the temperature of the T-ribs was the highest, followed by the temperature at the horizontal and longitudinal partitions, and the temperature of the concrete was the lowest. Although the steel structure heated rapidly in a fire, its effect on the overall tunnel temperature was relatively limited, and the impact of the steel components inside the tunnel on the overall tunnel temperature was small.
(2) The warming of the tunnel tube section structure was concentrated within 30 cm of the outer surface of the steel shell. Beyond this range, the temperature was close to the ambient temperature.
(3) Without fire protection measures for the tunnel tube structure, the surface temperature of the steel shell could exceed the NFPA 502 fire protection temperature of 300 °C for steel-tunnel-lining surfaces in as little as 500 s.
(4) There was a significant difference in temperature transfer between steel and concrete. The temperature of the steel section rose quickly and affected a considerable depth, while the temperature of the concrete section rose slowly and affected a small depth. The main effect of temperature on the steel structure of the tunnel under different types of vehicle fires was to a depth of 30 cm from the outer surface of the steel shell and on the concrete to a depth of 20 cm. The maximum difference between the temperature at the partition and the concrete was located 10 cm from the outer surface of the steel shell, and the maximum temperature difference was up to 149 °C.

## Figures and Tables

**Table 1:** Thermal parameters for materials.

Material	Density (kg/m^3^)	Specific Heat Capacity (kJ/(kg·K))	Thermal Conductivity (W/(m·K))
Steel	7850	0.46	45.8
Concrete	2400	1.04	1.8
Fireproof board	1086	0.8	0.242

**Table 2:** Setting of simulated scenarios.

Abbreviation of Conditions	Type of Fire	Peak Heat Release Rate (MW)	Whether have the Fireproof Board
1-minibus	1 minibus spontaneously combusted	8 MW	false
			true
1-bus	1 bus spontaneously combusted	30 MW	false
			true
1-van	1 van spontaneously combusted	15 MW	false
			true
2-minibus	Collision of 2 minibuses	16 MW	false
			true
3-minibus	Collision of 3 minibuses	24 MW	false
			true
5-minibus	3 minibuses set fire to 2 minibuses	40 MW	false
			true

**Table 3:** Working conditions’ settings.

Abbreviation of Conditions	Type of Fire	Peak Heat Release Rate (MW)	Has the Fireproof Board
HC	-	-	false
			true
1-minibus	1 minibus spontaneously combusted	8 MW	false
			true
1-bus	1 bus spontaneously combusted	30 MW	false
			true
1-van	1 van spontaneously combusted	15 MW	false
			true
2-minibus	Collision of 2 minibuses	16 MW	false
			true
3-minibus	Collision of 3 minibuses	24 MW	false
			true
5-minibus	3 minibuses set fire to 2 minibuses	40 MW	false
			true

## Data Availability

The original contributions presented in this study are included in the article. Further inquiries can be directed to the corresponding author.
